# A rare case of rectal cancer with perianal metastasis: a case report

**DOI:** 10.1186/s12957-019-1692-7

**Published:** 2019-08-20

**Authors:** Takuto Ikeda, Atsushi Nanashima, Akiko Ichihara, Eiji Kitamura, Kenzo Nagatomo, Hiroyuki Tanaka

**Affiliations:** 1 Division of Gastrointestinal, Endocrine and Pediatric Surgery, Department of Surgery, Faculty of Medicine, University of Miyazaki, 5200 Kihara, Kiyotake-cho, Miyazaki City, Miyazaki, 889-1692 Japan; 2 Division of Hepato-Biliary-Pancreas Surgery, Department of Surgery, Faculty of Medicine, University of Miyazaki, City, Miyazaki, Japan; 3 Section of Oncopathology and Regenerative Biology, Department of Pathology, Faculty of Medicine, University of Miyazaki, City, Miyazaki, Japan

**Keywords:** Metastatic anal tumor, Anal fistula, Colon cancer

## Abstract

**Background:**

Cancer metastasis from colon cancer to an anal fistula is very rare. We herein reported a rare case in which local excision was performed for metastatic anal fistula cancer originating from rectal cancer.

**Case presentation:**

A 68-year-old man was referred to our institution with a diagnosis of rectal cancer. He had complained of anal fistula for 5 years. Based on a recent history of cerebral infarction, Hartmann’s operation was performed to treat the rectal cancer after the administration of preoperative chemotherapy for 3 months. However, 1 month after Hartmann’s operation, the anal fistula was found to have worsened. Pelvic magnetic resonance imaging (MRI) revealed tumor formation at the perianal lesion. Metastatic anal fistula cancer originating from the rectal cancer was diagnosed based on the examination of the biopsied tissue. We selected local excision because the anal tumor had not invaded the surrounding tissue. There has been no recurrence in the 31 months after the curative operation.

**Conclusion:**

Metastatic cancer should be ruled out when treating left-sided colon cancer with anal fistula. Local excision is one possible treatment for metastatic anal fistula cancer.

## Background

One of the reasons for local recurrence at the anastomotic site after colectomy is thought to be due to the implantation of exfoliated cancer cells in the raw mucosa during anastomosis. It is thought that intraluminal exfoliated cancer cell implantation into the normal colorectal mucosa never happens without a mucosal defect. However, cancer cell implantation may be induced in an anal fistula due to mucosal destruction resulting from chronic inflammation. Rare cases of metastatic anal fistula cancer from colorectal cancer have been reported in the literature, some of which were treated by abdomino-perineal resection [1–11]. We herein present a very rare case of locally resected metastatic anal fistula cancer originating from rectal cancer, which showed a good prognosis.

## Case presentation

**Fig. 1:**
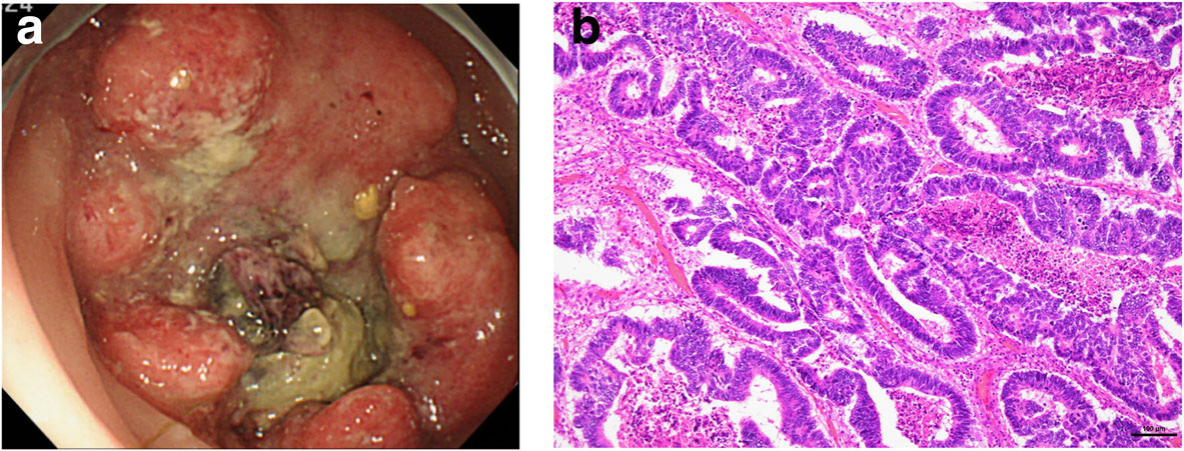
**a** Colonoscopy revealed a type 2 tumor at the recto-sigmoid colon. **b** The surgically resected rectal specimen showed columnar or polygonal cells with hyperchromatic nuclei proliferating in tubular and cribriform patterns. Well-differentiated tubular adenocarcinoma was diagnosed

**Fig. 2:**
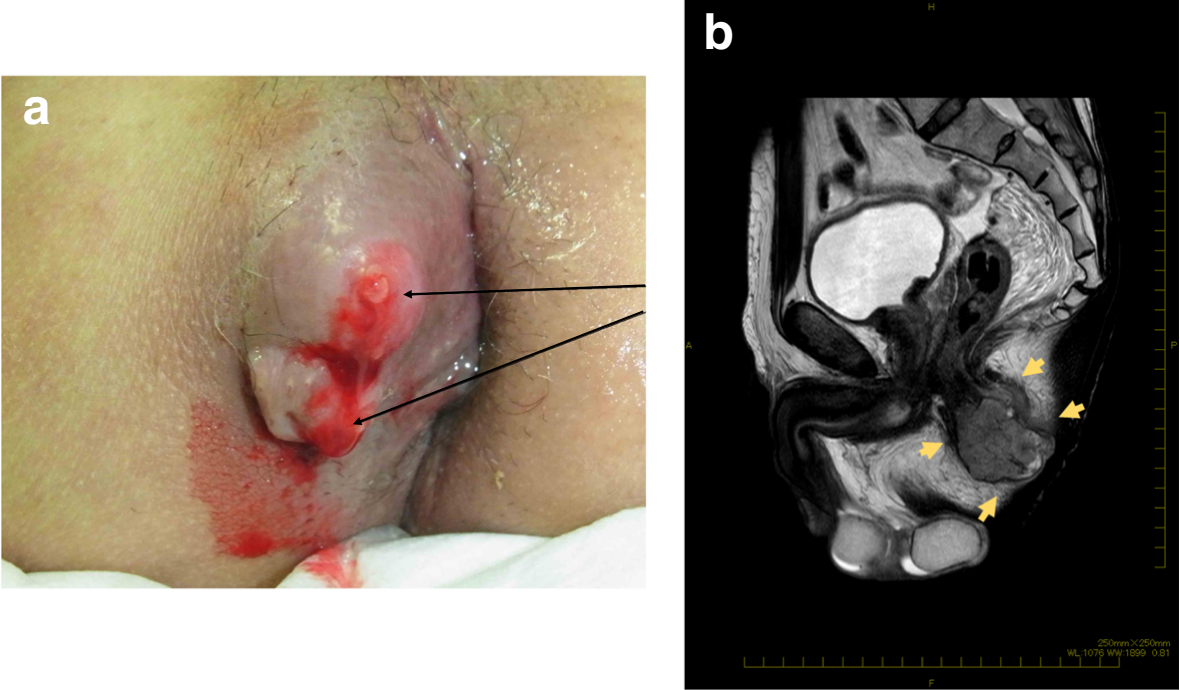
**a** An elastic hard tumor with two secondary openings (arrows) was detected at the right side of the anus. **b** MRI T2-weighted imaging revealed a heterogeneous high-intensity tumor of 4.5 cm in size at the perianal region (arrows)

**Fig. 3:**
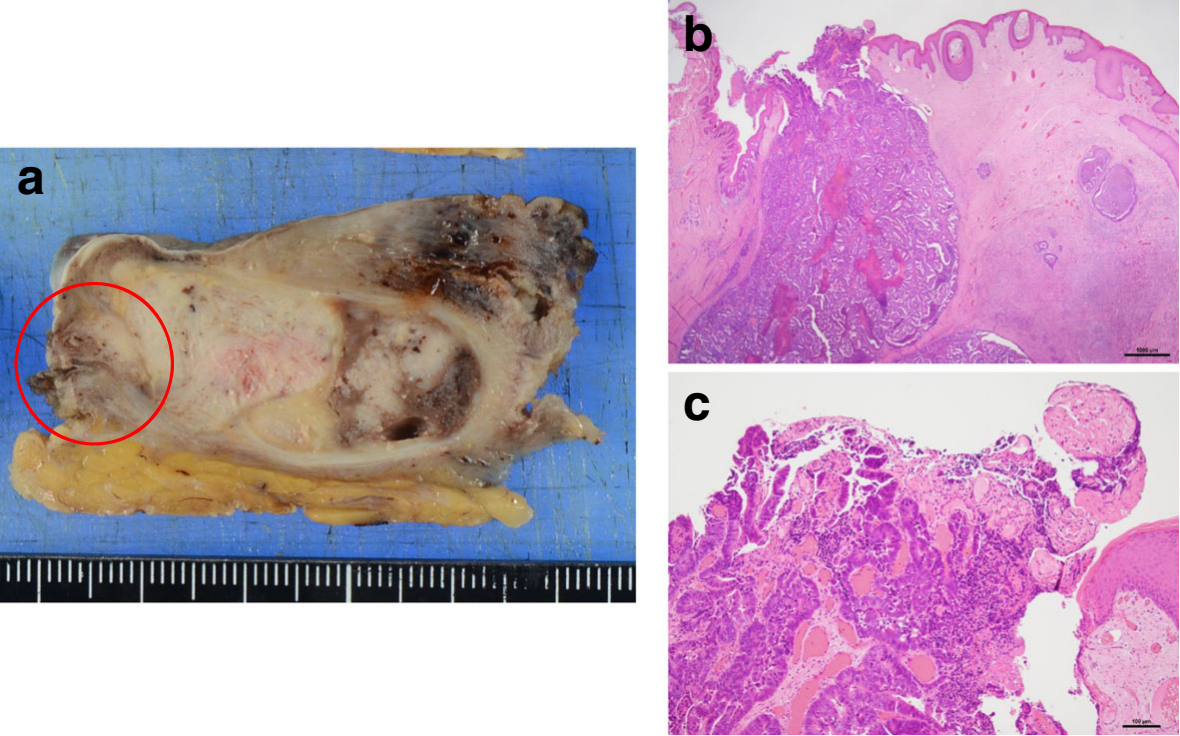
**a** A resected specimen of the anal tumor. Macroscopically, the tumor grew in a capsulized cavity without invasive growth. The pathological findings of the encircled region in panel **a** are shown in panels **b** and **c**. **b** Tumor growth without invasion to the surrounding tissue is detected. The apex of the tumor is exposed at the secondary opening (× 12.5). **c** Atypical epithelial cells with hyperchromatic nuclei and notable nuclei proliferate in a tubular pattern. Metastatic adenocarcinoma from rectal cancer was considered (× 100)

**Fig.4:**
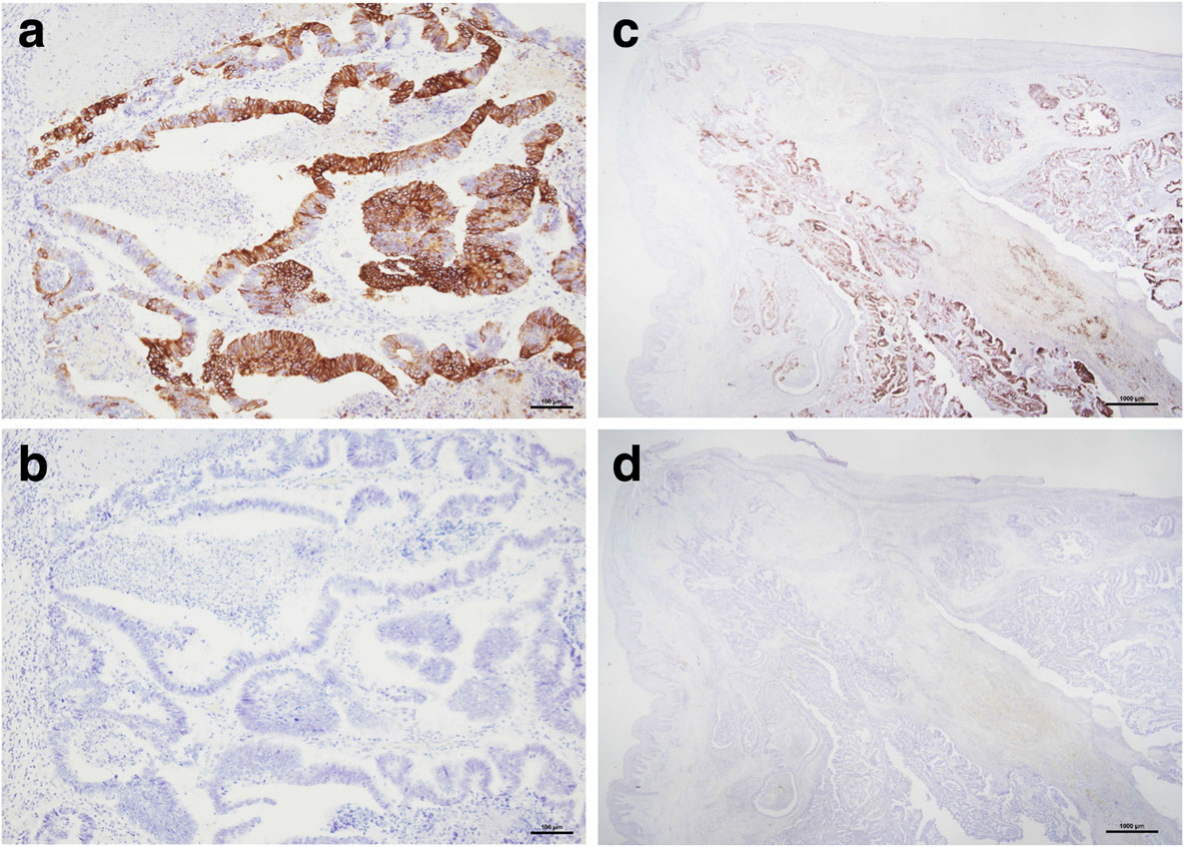
**a** Sections of the rectal tumor were immunopositive for cytokeratin 20 (× 100) and **b** immunonegative for cytokeratin 7 (× 100). Sections of anal fistula tumor were **c** immunopositive for cytokeratin 20 (× 12.5) and **d** immunonegative for cytokeratin 7 (× 12.5)

A 68-year-old man was referred to our institution with a diagnosis of rectosigmoid colon cancer. Colonoscopy revealed a type 2 tumor at the rectosigmoid colon (Fig. 1a). Right hemiparesis was present as a result of two cerebral infarctions that had occurred during the 2 months prior to his first admission. After consultation with a neurologist, we considered that it was necessary to wait several months before performing a major operation due to the risk of inducing another cerebral infarction. However, advanced cancer would be expected to grow during the waiting period. We therefore considered that it was necessary to administer systemic chemotherapy to suppress tumor growth. We initially administered five courses of preoperative chemotherapy (mFOLFOX6). No cerebral events or severe adverse events occurred during the chemotherapy. Stable disease, according to the RESIST criteria, was achieved with the preoperative chemotherapy. A laparoscopic Hartmann’s operation with D3 lymph node dissection was performed at 4 weeks after the last course of chemotherapy. The patient’s postoperative course was good without any complications. The final diagnosis of the rectal cancer was Dukes B with well-differentiated tubular adenocarcinoma and rectal tumor invasion to the subserosa (T3) without lymphatic or venous invasion (Fig. 1b). The resected specimen was free from cancer cells. The patient had complained of a perianal abscess for 5 years before his first admission. However, we gave priority to treating the rectal cancer. Although his anal symptoms did not worsen during chemotherapy, at 1 month after the Hartmann’s operation for rectal cancer, his perianal abscess worsened and induration with two secondary open lesions was detected on physical examination (Fig. 2a). The pathological examination of the biopsied perianal tissue revealed adenocarcinoma. Because the perianal tumor was localized without invasion of the neighboring tissue on magnetic resonance imaging findings (Fig. 2b), and considering his physical states, we performed local excision under spinal anesthesia for curative resection. The final pathological diagnosis of the resected specimen was metastatic adenocarcinoma to an anal fistula originating from rectal cancer, and the resected margin was free from cancer cells (Fig. 3a–c). Immunohistochemistry revealed that both the rectal tumor and anal tumor were cytokeratin 7 (CK7) − and cytokeratin 20 (CK20) + (Fig. 4). Based on the pathology of the primary tumor and the absence of lymphatic or venous invasion, metastasis was considered to have occurred due to the implantation of exfoliated cancer cells. Seven courses of postoperative adjuvant chemotherapy with the same regimen have been administered. No recurrence of the tumor has been seen for 31 months after the operation. If a locally recurrent tumor had been found near the rectum and anus without distant metastasis during follow-up, laparoscopic resection of the tumor with the residual rectum and anus would have been attempted after considering the patient’s performance status.

## Discussion

Metastasis from colorectal cancer to an anal fistula is very rare. The mechanism is thought to involve the adherence of free cancer cells to the tract of the anal fistula followed by tumor proliferation and invasive growth. Destruction of the intestinal mucosa may induce the adherence of cancer cells. Mekata et al. reported that damage to mucosal sites from obstructive colitis could induce the luminal implantation of cancer cells [12]. Hubens et al. revealed that mucosal damage caused by biopsy in the presence of viable colon cancer cells resulted in mucosal implantation and intraluminal growth in one out of 30 rats [13].

In 1954, Guiss reported a case of implantation of cancer cells within an anal fistula from sigmoid colon cancer [1]. Since then, 22 cases of metastatic anal fistula cancer originating from colorectal cancer due to exfoliated cancer cell implantation have been reported in the English literature [2–11, 14–22]. We evaluated 24 cases, including our own (Table 1). All patients were males, and the tumors were located on the left-side colon, especially distal to the sigmoid colon. The period for which the anal fistula persisted before the diagnosis of the metastatic anal tumor ranged from 2 months to 20 years. Although most patients’ anal tumors were pointed out synchronously with primary cancer, in four cases, they were detected at more than 1 year after surgery for the primary tumor.

**Table 1:** Evaluation of 24 cases

	Gender	Age	Stage	Location	Operation	Sync or not	Chemo	Prognosis
Guiss [1]	M	63	DukesA	S	APR	Sync	None	1 year, 2 months (-)
Killingback et.al [2]	M	47	DukesA	S	APR	NA	NA	NA
Parnes [3]	M	65	DukesB	S	APR	Sync	None	18 months (-)
Rollinson and Dundas [4]	M	68	NA	Rs	APR	Sync	None	10 months (-)
Thomas and Thompson [5]	M	36	DukesB	S	APR	Sync	NA	NA
Isbister [14]	M	39	DukesC	S	Hart+not rese	12 months later	None	NA
Isbister [14]	M	47	DukesC	Rs	AR+not rese	12 months later	None	NA
Isbister [14]	M	69	DukesC	Rs	By-pass	Sync	None	NA
Shinohara et al. [15]	M	66	DukesC	RaRb	LAR+LR	Sync	NA	6 months liver meta
Kouraklis et al. [6]	M	47	DukesB	S	APR	Sync	NA	NA
Hyman and Kida [7]	M	44	DukesB	S	APR	Sync	None	1 year (-)
Zbar and Shenoy [16]	M	72	NA	S	S+LR	Sync	CRT	1 year, 2 months (-)
Gupta et al. [17]	M	53	DukesC	Left colon	Lt. colec+LR	Sync	5FU/LV	3 years (-)
Hamada et al. [18]	M	53	DukesB	S	AR + LR (coring out)	Sync	UFT/UZEL	1 year (-)
Ishiyama et al. [19]	M	53	DukesC	Ra	AR + LR	Sync	NA	10 months died with carcin
Sandiford et al. [20]	M	72	DukesB	Rs	S+LR	Sync	CRT	14 months (-)
Gravante et al. [8]	M	64	DukesA	Left colon	Lt. colec, APR	13 months later	CRT	1 year (-)
Wakatsuki et al. [21]	M	57	DukesB	Rs	AR+LR	15 months later	None	3 years, 7 months (-)
Takahashi et al. [11]	M	61	NA	S	APR	Sync	5FU/LV/CPT11	36 months (-)
Benjelloun et al. [22]	M	68	DukesB	Rs	AR + LR	Sync	NACRT→CR	3 years (-)
Benjelloun et al. [22]	M	55	DukesB	Rs	AR + LR	Sync	NACRT→CR	3 years (-)
Gomes et al. [10]	M	65	DukesB	S	APR	Sync	None	3 months (-)
Takahashi et al. [11]	M	80	DukesC	Ra	Lap APR	Sync	FOLFOX6	14 months (-)
Our case	M	68	DukesB	Rs	Hart+LR	Sync	FOLFOX6	28 months (-)

*APR* abdomino-perineal resection, *Hart* Hartmann, *not resect* not resected, *AR* anterior resection, *LR* local resection, *CRT* chemoradiation, *NACRT* neoadjuvant chemoradiotherapy, *Lt. colec* left colectomy, *Lap* laparoscopic, *sync* synchronous, *S* sigmoid colon, *Rs* recto-sigmoid colon, *Ra* upper rectum, *Rb* lower rectum, *NA* not available

When making a diagnosis of metastatic anal fistula, we must first rule out primary anal fistula cancer. Many cases were diagnosed based on the findings of hematoxylin-eosin staining. Moreover, as was used in our case, immunohistochemical staining of CK7 or CK20 has been used for differentiation in many cases [7, 8, 10, 11, 17, 18, 21, 22].

Radical resection of the primary tumor combined with the metastatic tumor has a crucial role in the treatment of metastatic anal fistula. Curative resection was performed in 21of 24 cases. Abdominoperineal resection (APR) was performed for 11 of 24 patients (45.8%). On the other hand, including our case, 10 patients (41.6%) were treated with local resection of the anal tumor as an anus-preserving curative operation [15–22]. Among 10 patients, 7 patients underwent perioperative chemotherapy or chemoradiotherapy. All of these patients showed a good prognosis without recurrence. Local resection can be selected for a localized anal fistula tumor with no invasive growth.

There is some debate as to whether surgery should be performed for primary cancer and metastatic anal cancer at the same time or at a different time. We are of the opinion that surgery for the primary rectal cancer should be performed first. If we had operated on the anal fistula first, the anal wound could have induced local recurrence due to the migration of exfoliated cancer cells from the primary rectal cancer. Simultaneous procedures might induce local recurrence at the perianal surgical wound. Moreover, patients with anal tumors must be carefully observed for re-recurrence for 1 to 2 years after a radical operation. During the operative procedure for the anal tumor, care must be taken to prevent the migration of cancer cells into the incision. During our patient’s operation, we covered the anal tumor with gauze, which was sutured to the skin around the tumor.

The prognosis in these advanced cases is not so severe, with many of the reported patients surviving without recurrence. Perioperative adjuvant and neoadjuvant therapy, which is indicated in some cases, may improve the prognosis. Furthermore, a metastatic route that does not involve lymphovascular invasion may be another reason for the good prognosis. Knowledge of the initial symptom related to a perianal tumor may contribute to the early diagnosis of colon cancer. In many of the reported cases, the follow-up time was inadequate. Thus, the accumulation of additional data from cases with long-term follow-up is needed.

## Conclusion

We presented a very rare case of a metastatic anal fistula tumor originating from colon cancer. Treatment for colorectal cancer in patients with an anal fistula must be performed in consideration of the possible development of a metastatic anal fistula tumor. Although an extended operation leaving no residual tumor is an important means of treatment, local resection should not be excluded for non-invasive tumors or in cases involving patients with severe complications.

## Data Availability

All data generated or analyzed are included in this published article.
